# Negative feedback concept in tagging: Ghost tags imperil the long-term monitoring of fishes

**DOI:** 10.1371/journal.pone.0229350

**Published:** 2020-03-02

**Authors:** Marek Šmejkal, Daniel Bartoň, Vilém Děd, Allan T. Souza, Petr Blabolil, Lukáš Vejřík, Zuzana Sajdlová, Milan Říha, Jan Kubečka

**Affiliations:** Biology Centre of the Czech Academy of Sciences, Institute of Hydrobiology, České Budějovice, Czech Republic; Institut de recherche pour le developpement, FRANCE

## Abstract

Wildlife monitoring using passive telemetry has become a robust method for investigating animal migration. With increased use, this method progressively pollutes the environment with technological waste represented by so called ghost tags (PIT tags ending in the environment due to reproductive expulsions, shedding or animal mortality). However, their presence in the environment may lead to failed detections of living individuals. We used tagging data from studies of the asp *Leuciscus aspius* and the bleak *Alburnus alburnus* collected from 2014 to 2018 and located ghost tag positions on the monitored spawning site using portable backpack reader for their detection. We modelled virtual river-wide flat-bed antennas (widths 0.2, 0.4, 0.6 and 0.8 m) representing monitoring effort and estimated the probability of the presence of ghost tags within the antenna field. Of 3724 PIT tags used in the study, we detected on the spawning ground 173 ghost tags originating from long-term monitoring. The ghost tags accumulated in the environment in time, suggesting insufficient degradation rate or shift downstream from the research site. Number of ghost tags present on the spawning ground led to high probability of disabled readings of tagged fish passing through the antenna electro-magnetic field. We demonstrate how accumulated ghost tags may cause detection failures for focal species and incomplete data acquisition. We infer that intensive long-term monitoring using PIT tag technology may encumber future data acquisition or entail additional costs for clean-up.

## Introduction

Over the past century, waste production has become increasingly problematic, in particular anthropogenic wastes that are toxic and/or persistent in the environment [1–3]. Environmental recovery from anthropogenic pollution is often difficult and expensive [4]. Scientific research is not usually seen as an important source of contaminants but can in fact contribute to waste pollution [5]. Awareness of the possible impacts of laboratory waste is long established and many disposal programmes are currently in place to reduce these environmental impacts [6]. On the other hand, wildlife tracking systems are relatively new tools that, due to technological advancement, have become cheaper and frequently integrated into environmental monitoring programmes around the world [7,8]. These systems generate abundant data on species movement, allowing ecologists to track the behavioural dynamics of animals of different taxa, yielding data with unprecedented resolution [9,10]. Despite the benefits of this novel tool, virtually nothing is known about the fate of these tracking devices when the animal loses them or dies [7].

Radio frequency identification technology (RFID) for animal passive telemetry has enabled monitoring of individuals and populations for long periods, with potential to assess dispersal and migration in defined pathways [11–15]. The principle of the PIT-tag reading is that PIT-tag is charged by the electro-magnetic field created in the antenna loop and when fully charged, it emits a unique code that identifies given individual. The obvious advantage is that an animal does not have to be captured or observed directly by a scientist/wildlife manager, since the passive integrated transponder (PIT tag) located in the animal's body can be automatically scanned by an antenna loop installed on the migration route [16–18]. PIT tags do not require a battery, which greatly reduce their size and enables to tag relatively small animals [19,20]. This system has enabled automatic collection of migratory data, limited mainly by the width of the migratory pathway and our ability to define places on the migration route that can be covered by the antenna scanning range [21,22].

PIT tags are certain to end up in the environment due to several issues. PIT tags are released into the environment by dead individuals due to their natural mortality. These PIT tags are retained in rivers for long periods and may represent a substantial source of false positive detections of fish that are already dead [23–25]. Fish reproduction can result in PIT tag loss from an individual due to expulsion of gametes [26,27] and fish can also shed them through insertion site [28]. Both expelled and mortality related PIT tags are referred to in the scientific literature as "ghost tags" [23,29]. Ghost tags that accumulate at a study site within antenna range may interfere with effective detection of migrating individuals due to the inability of the system to read more than one PIT tag at a time—so called "PIT tag collisions" [30–32]. Although indefinite lifetime is one of the major advantages of PIT tags, in the case of ghost tags their long life can become disadvantageous and a source of scientific waste in the environment [23]. If the goal of a study is long-term monitoring, the presence of ghost tags may cause inefficient monitoring and antenna underperformance.

The aim of this study was to evaluate whether PIT tag pollution can potentially complicate construction of river-wide antennas and how this phenomenon may affect future monitoring designs. We used adaptive monitoring data on 3724 fish (asp *Leuciscus aspius* and bleak *Alburnus alburnus*) from 2014 to 2018 to demonstrate the effect of monitoring waste on monitoring efficiency. The study emphasizes the potential importance of PIT tag pollution on research sites and the potential for unexpected problems in long-term monitoring studies due to ghost tag pollution clean-up or necessary changes in technology.

## Materials and methods

### Study site

**Fig 1:**
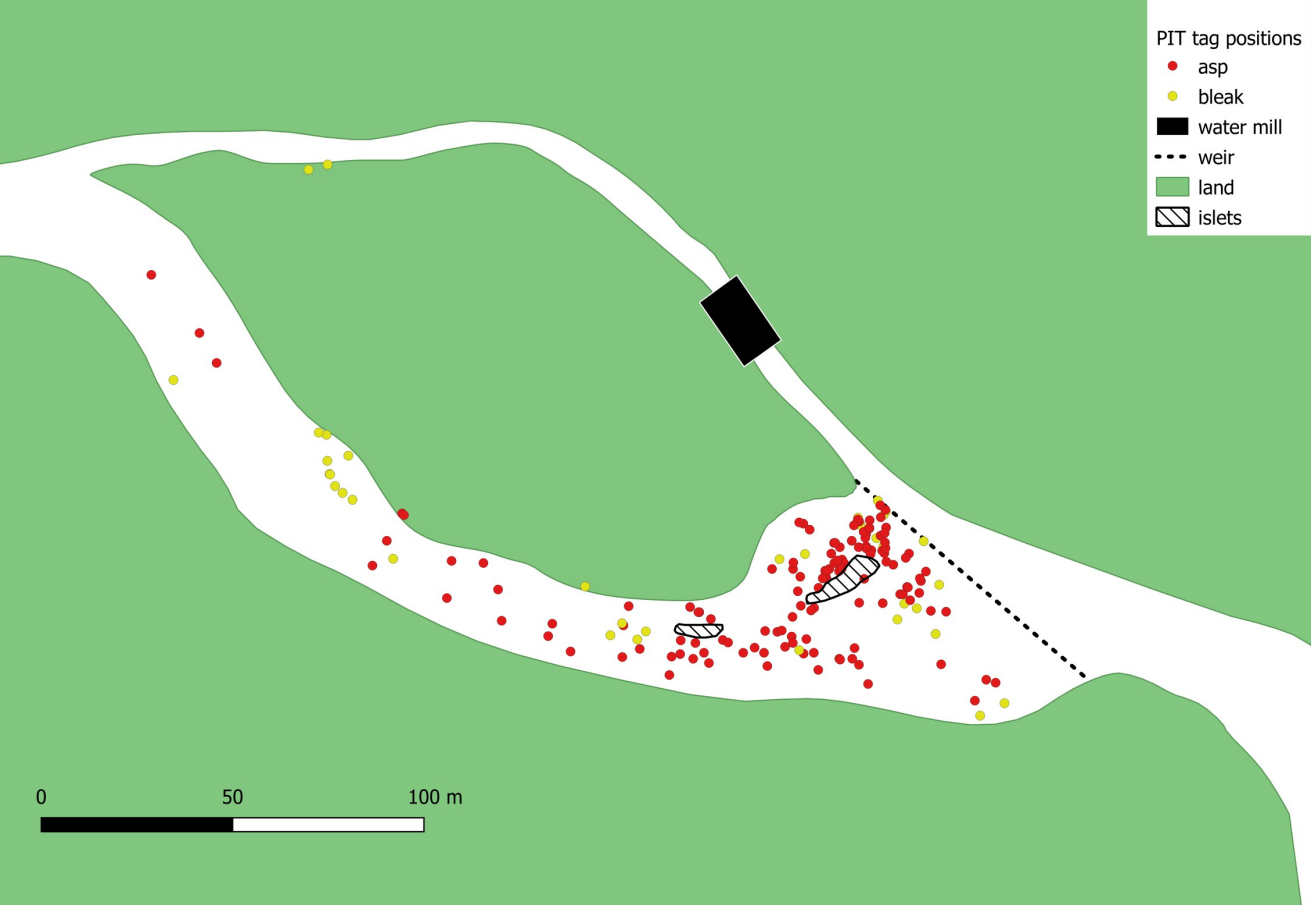
Schematic visualization of the research site and its close surroundings showing the GPS positions of asp (red dots) and bleak (yellow dots) ghost tags detected by portable antenna. Further upstream migration is restricted by a weir and spawning typically occurs in the most fluvial portion of the river closest to the weir. The arrow shows the direction of flow. The islets visualized may be partially flooded during the spawning period.

The research was conducted in the Želivka River (latitude 49°34'42"N, longitude 15°15'14"E), the main tributary of the Želivka Reservoir, containing the spawning ground of the reservoir's asp population (approximately 2000 adult individuals) [33]. Asps are a long-lived iteroparous species that reaches maturity in the fourth to sixth year of life, returning to river spawning grounds yearly [34,35]. Adults in the studied population are typically 45–85 cm in total length (TL). They enter fast flowing water (0.2–0.4 m.s^-1^) for reproduction in the early spring. The spawning ground is located close to a weir (Fig 1). Eggs released by females adhere to stones and pebbles. The bleak is a short-lived small cyprinid species that enters the spawning ground together with the spawning asps and feeds on their eggs [34]. These fishes' migration is restricted by the weir, which limits their spawning ground to an approximately 100 m long stretch of the river [35].

### Fish capture and tagging procedure

Fish were captured using an electrofishing boat (Electrofisher EL 65 II GL DC, Hans Grassel, Schönau am Königsee, Germany, 13 kW, 300/600 V) during the spawning seasons of 2014 to 2018 (mid-March to mid-April). Before the tagging procedure started, each fish was anaesthetized with MS-222, and their total length and weight were recorded. A 3–5 mm vertical incision was made on the side of the body and a PIT tag was inserted by hand into the body cavity. We used a 32 mm long PIT tag for adult asps (Oregon RFID, half-duplex, diameter: 3.65 mm, weight: 0.8 g, ISO 11784/11785 compatible) and a 23 mm long PIT tag for bleaks and juvenile asps (Oregon RFID, half-duplex, diameter: 3.65 mm, weight: 0.6 g, ISO 11784/11785 compatible). Tag functionality was verified with a hand reader (Oregon RFID, Easy Tracer II FDX/HDX Reader).

### Ethics

The field sampling and experimental protocols used in this study were performed in accordance with the guidelines and permission of the Experimental Animal Welfare Commission under the Ministry of Agriculture of the Czech Republic (Ref. No. CZ 01679). All methods were approved by the Experimental Animal Welfare Commission under the Ministry of Agriculture of the Czech Republic.

### Detection of ghost tags

We used HDX portable backpack reader (Oregon RFID, Portland, Oregon) for detection of ghost tags on the spawning ground. The scanning was conducted on 6 and 22 November 2018 when the water level was low (0.1–0.5 m), enabling us to sweep the antenna close to the river bottom [12]. We carried a GPS device (GPSmap 60 CSx, Garmin, USA, Kansas, Olathe) together with a portable antenna (50 cm diameter and reading ranges of 49 and 77 cm for parallelly and perpendicularly oriented PIT tags, respectively) to record the position of the ghost tag, merging time of detection with position at that time. The time settings of both devices were synchronized. During the survey we visually inspected the water to ensure that no fish were present at the locations of ghost tag detections. Both asp and bleak are open-water species [36] with no tendency to hide among stones.

### Assessing the probability of tag collisions between ghost tags and tagged fish

The GPS positions of ghost tags were established by mapping of the spawning ground using the portable antenna. The time of first ghost tag detection was used to establish the ghost tag's GPS position. A line was drawn in the centre of the river and virtual river-wide antennas were constructed perpendicular to this line with a spacing of 0.1 m. We defined four reading ranges for the antennas: 0.2, 0.4, 0.6 and 0.8 metres. The number of ghost tags in the antennas depending on their position and reading range were computed from the model.

### Statistical analysis

A linear regression was used to test the dependence of the number of ghost tags on the year of the monitoring programme. The dependence of the ghost tag count on the smoothed (cubic regression splines with shrinkage) distance from the weir was assessed using a generalized additive model (GAM) fitted using a Poisson distribution with grouping on the different antenna detection ranges (0.2, 0.4, 0.6, 0.8 m) [37]. Ghost tags from both species and life stages were pooled together for the analysis. The analysis and plotting of the fitted values of GAM were conducted using R software version 3.4.3 [38]. QGIS software was used for the graphical presentation of ghost tags on the spawning ground [39].

## Results

**Fig 2:**
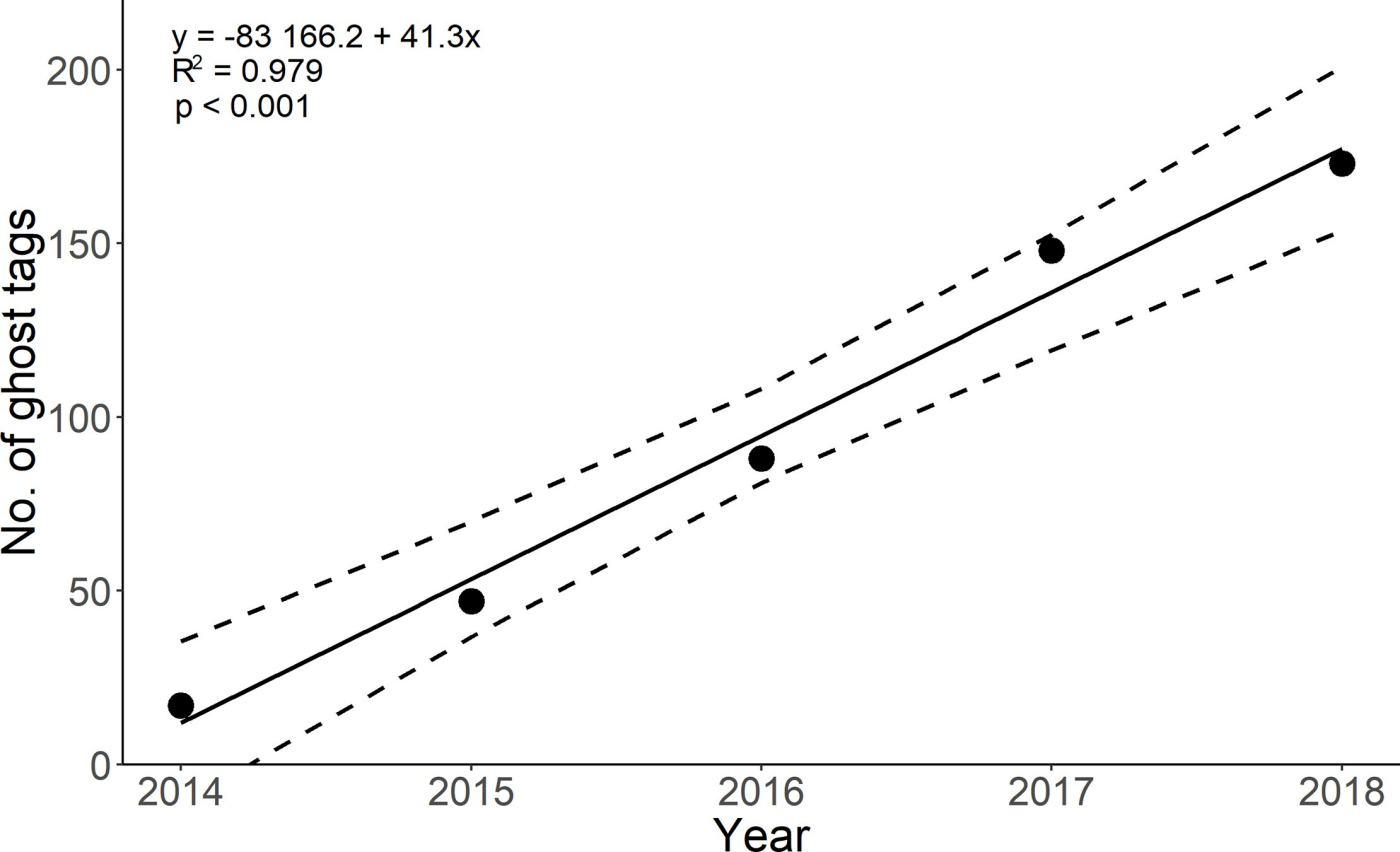
Accumulation of ghost tags on the spawning ground during the five years of the monitoring study. The points represent the cumulative number of PIT tags identified by portable backpack antenna. Dashed lines represent confidence intervals.

In the five years of the monitoring programme a total of 2318 adult asps (mean TL 58.0 cm ± 4.9 cm standard deviation; size range 37.5–795 cm) and 303 juvenile asps (21.3 cm ± 12.0 cm; 12.8–52.5 cm) and 1103 bleaks (14.8 cm ± 10.8 cm; 11.5–19.3 cm) were tagged. Of PIT tags used in the study, we detected on the spawning ground 129 ghost tags originating from adult asps, 6 ghost tags originating from juvenile asps and 38 ghost tags originating from bleaks (Table 1, Fig 1). The number of ghost tags was dependent on the year of monitoring, suggesting accumulation of the ghost tags in the environment and insufficient degradation or shift downstream from the research site (F1, 3) = 187.9, p< 0.001, R^2^ = 0.979; Fig 2).

**Table 1:** Number of implanted tags in 2014 to 2018 (No. of T) and number of ghost tags (GT) identified on the spawning ground from each tagging season. Adult and juvenile asps are presented separately due to different mechanisms of ghost tag production (absence of spawning behaviour).

Tagging year	No. of		No. of		No. of		No of.		
	T	GT	T	GT	T	GT	T	GT	%
2014	355	17	24	0	0	0	379	17	4.5
2015	391	30	6	0	0	0	397	30	7.6
2016	617	35	8	0	222	6	847	41	4.8
2017	587	31	241	6	514	23	1342	60	4.5
2018	368	16	24	0	367	9	759	25	3.3
Total	2318	129	303	6	1103	38	3724	173	4.6

**Fig 3:**
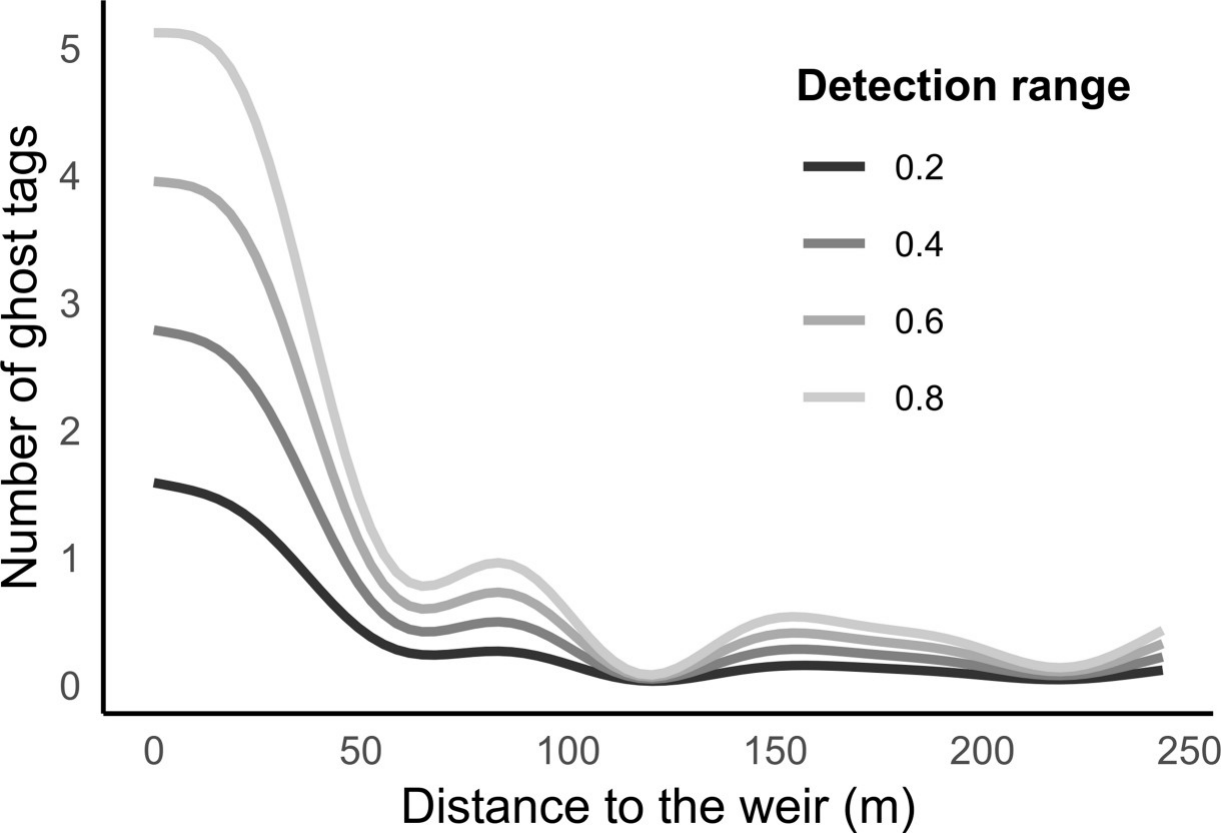
The GAM model assessing the number of ghost tags within antenna detection range related to distance from the weir. The ghost PIT presence in the antenna reading range can cause reading collisions and an inability to detect living individuals.

The GAM model demonstrated that the number of ghost tags was much higher in the first 50 metres from the weir (asp major spawning ground), suggesting a high probability of PIT tag collision, especially with an antenna design with a high reading range. After the first 50 metres, the number of ghost tag collisions decreased rapidly, and differences between the detection ranges were minor (Table 2, Fig 3).

**Table 2:** Generalized additive model (GAM) summary table. edf = estimated degrees of freedom. Ref.df = reference number of degrees of freedom used for hypothesis testing.

Parametric coefficients	Estimate	SE	z-value	p-value
Intercept	-0.92	0.02	-47.68	<0.001
Approximate significance of smooth terms	edf	Ref.df	χ^2^	p-value
Detection range 0.2 m	6.59	9	993.1	<0.001
Detection range 0.4 m	7.96	9	2610.2	<0.001
Detection range 0.6 m	8.34	9	4482.3	<0.001
Detection range 0.8 m	8.53	9	6462.7	<0.001
R^2^(adjusted)	0.55			
Deviance explained (%)	52.3			

## Discussion

This study demonstrates that long-term monitoring of the research site may not be constantly effective over time due to increasing impact of the ghost tags present in the environment. Specifically, we found a positive relationship between the duration of the study and ghost tags detected in the environment. Our study suggests that the abundance of ghost tags may prevent effective monitoring with flatbed antennas after just five years of monitoring. We infer that long-term research monitoring using passive telemetry often does not allocate funds for removing PIT tags from the environment, which may decrease the efficiency of the research and imperil the continuity of monitoring programmes, forcing scientists and stakeholders to change their monitoring schemes.

The presence of ghost tags can be attributed to shedding and fish mortality [23,26,40,41]. Reproductive expulsion of PIT tags is common and seems to be a very important contributor to the observed accumulation of ghost tags. We recorded a retention rate of 85 to 98% in asps, while in salmonids the retention rate during reproduction is often lower (65–92%) [27,41–45]. Some salmonid fishes are semelparous and PIT tags used for tracking end up in the environment after their deaths [23]. Given that this technology is more frequently used to monitor salmonids than cyprinids, it is possible that the effects of ghost tag accumulation may more heavily impact critical areas for salmonid reproduction [23]. In our study, most of the ghost tags from bleaks and juvenile asps are likely to be result of the natural mortality and predation by predators such as northern pike, *Esox lucius*, and herons, *Ardea cinerea*, whereas ghost tags originating from adult asps come mainly from spawning activity [41].

Whatever the origin of ghost tags, their accumulation on the research site may be significant and results in difficulties for antenna deployment or in hindered data acquisition by passive telemetry systems. Our results showed that after 5 years of monitoring we cannot deploy river-wide antennas closer than 50 metres from the spawning ground if we wish to avoid substantial numbers of collisions. Antenna systems may be fully functional following their installation at the beginning of the season yet experience declining efficiency due to new ghost tag accumulation during the monitored season, with subsequent PIT tag collisions [30–32]. For this reason, the assumption that the probability of detecting PIT tags is equal throughout the course of a spawning season can be questioned [46]. For instance, fish activity measured by number of detections per time period can be influenced due to a lower probability of detection late in the reproductive season, when expelled PIT tags have accumulated in the environment and prevent detection of tagged fish [18,35]. This is in conflict with the actual purpose of many long-term studies, where the same sampling scheme (and therefore antenna positions) should be maintained to compare data between years [47,48].

The asp spawning ground, where the study was conducted, is of limited size due to the weir, which blocks the upstream migration of large fish such as asps. The spawning ground extends approximately 100 m depending on water level and velocity in given year [35]. The effect of PIT tag loss is enhanced by the presence of PIT tags from bleaks, used during adaptive monitoring of the asp spawning ground in later years [34]. Since our research site is limited to such a small area, within which all fish enter, reproduce or are predated, the effect of ghost tag pollution may be observed over a relatively short monitoring programme. Larger systems may be monitored longer without serious ghost tag issues, but eventually ghost tag pollution may become problematic in any long-term monitored system.

Since many streams are now monitored for prolonged periods of time, there is an increasing need to investigate the destiny of ghost PIT tags and their potential negative influence on scientific data [23]. In slow flowing streams where the substrate consists of relatively fine particles such as sand and gravel, PIT tags are transported downstream as much as several hundred metres and may not be problematic if monitoring focuses on fixed locations, but these transported PIT tags can result in false detection of fish that are no longer living but appear to be moving downstream from the monitored portion of the river [23]. In the rocky substrate ghost tags can potentially remain in crevices among the stones for an extended time. Our research site contains five-year-old ghost tags used at the beginning of the monitoring programme.

While fish swimming close to the water surface can be monitored using antennas deployed a minimum of several centimetres above the substrate to avoid collisions with ghost tags, demersal and benthic species must be monitored with flatbed antennas or antenna located very close to the bottom [49,50]. In such cases, the solution may be to shift antennas away from reproductive sites and restrict monitoring of reproduction to presence/ absence data. This has the obvious disadvantage of hindering comparison of the activities of individual fish on the reproductive site [18]. Collecting expelled PIT tags is only a temporary solution, since many may be deposited each season depending on sample sizes. PIT tags are very difficult to find in a fast-flowing river and some of them are buried in the substrate [23]. In cases where a spawning ground is of limited size, researchers might try using 12 mm PIT tags injected into muscles, with an approximately 99% retention rate [45], with the goal of decreasing generation of ghost tags by reproduction.

PIT tag technology use is not restricted to aquatic environments and fish ecology: they are increasingly popular in the study of invertebrates and of other vertebrates [15,19,51–54]. PIT tags have even been applied to monitoring the dynamics of abiotic environments, for example, marking stones and pebbles and tracking their movement in rivers [55]. Due to this breadth of application, research groups might not be always be aware of one another and the targets of one study may become the ghost tags for another, potentially reducing the amount and precision of data collected. Creating a global database, where source company would provide list of PIT tags along with user information, would help to identify owner of the PIT tag detected and potentially shed light on the species migration distances.

Waste production has increased with technological development and purchase power. Although wildlife monitoring still struggles to obtain funds for addressing key questions, budgets have undoubtedly increased in recent decades and the price of PIT tags has decreased from $4–7 to currently less than $2 [11]. Some studies use more than a hundred thousand PIT tags yearly for salmon monitoring [23]. Potentially problematic issues include the costs of mitigating the effects of ghost tags or switching to different technologies in polluted research sites. We have focused this study on scientific pollution, but biomonitoring eventually generates regular waste, the impact of which has not yet been investigated. In our research monitoring of 3724 fish we used 2.7 kg of PIT tags. In highly monitored watersheds such as Scott Creek in California, tens of kilograms are annually being released into streams within tagged fish [23].

RFID technology is specific in its ability to detect only one PIT tag at a time; therefore, the disabling effect of waste production on future monitoring may be specific to this technology. Other monitoring techniques (bird rings, active telemetry, GPS positioning) can have other potential side effects. This evaluation of PIT tag technology demonstrates how scientific waste accumulates in the environment, and although other monitoring techniques provide alternatives for future research on polluted sites, such substitutions do not deal definitively with waste production. Potentially, the goals of proposed studies and the quantities of PIT tags used should be justified and approved on the basis on the waste production in addition to other considerations, especially in the case of protected areas. An important goal for RFID manufacturers is to develop environmentally friendly clean-up technology or possibly a means of deactivating ghost tags on the monitoring site.

To conclude, we emphasize that the endless life of PIT tags can be perceived as a disadvantage, since ghost tags can make long-term monitoring of fish movement in streams less precise. We encourage scientists to evaluate risks connected with PIT tag pollution depending on project goals, species ecology and the characteristics of the study site (size, fish mortality and PIT tag reproductive loss) and adjust antenna design to prevent low quality data acquisition. Finally, even if the study site is so large that the impact of ghost tags will unlikely affect study design, scientists should be aware that by animal tagging they are inadvertently polluting the environment with unknown consequences for future monitoring. We believe that due to the issues relating to the use of RFID technology, the current technology should be used with caution to promote the sustainable continuity of wildlife monitoring programmes.
